# Bacterial culture positivity in placental basal plate tissue and associated factors among women with spontaneous preterm births at Mubende Regional Referral Hospital, Uganda: A cross-sectional study

**DOI:** 10.1371/journal.pgph.0007002

**Published:** 2026-10-29

**Authors:** Shem Kasiita, Marie Pascaline Sabine Ishimwe, Musa Kasujja, Theodore Nteziyaremye, Sara Liban, Bivako Racheal, Ronald Kakande, Innocent Bwire, G.R. Neel, Sharifa Namutebi, Ziraba Abdallah, Theoneste Hakizimana

**Affiliations:** 1 Department of Obstetrics and Gynecology, Kampala International University, Ishaka, Uganda; 2 Department of Pediatrics and Child Health, Kampala International University, Ishaka, Uganda; 3 Department of Sciences, University of Rwanda, Kigali, Rwanda; 4 Department of Microbiology, Kampala International University, Ishaka, Uganda; 5 Department of Foundations, Kampala International University, Ishaka, Uganda; PLOS: Public Library of Science, UNITED STATES OF AMERICA

## Abstract

Aerobic bacterial culture positivity in placental basal plate tissue and its associated maternal factors remain poorly described in Uganda. This study estimated the prevalence of culture positivity and identified associated factors among women with spontaneous preterm births at Mubende Regional Referral Hospital. We conducted a hospital-based analytical cross-sectional study among 383 women between 15 May and 15 September 2025. Placental basal plate tissue specimens were collected aseptically immediately after delivery and processed for aerobic bacterial culture. Sociodemographic, obstetric and clinical data were obtained using interviewer-administered questionnaires and medical record review. Associations were examined using multivariable logistic regression and reported as adjusted odds ratios (aORs) with 95% confidence intervals (CIs). Statistical significance was set at p < 0.05. Positive cultures were identified in 159 women, giving a prevalence of 41.5% (95% CI: 36.5–46.6). Culture positivity was associated with fever (aOR = 11.32, 95% CI: 4.89–26.19), abdominal pain or uterine tenderness (aOR = 9.13, 95% CI: 4.37–19.08), membrane rupture lasting more than 24 hours (aOR = 5.93, 95% CI: 2.08–16.89), HIV infection (aOR = 4.23, 95% CI: 1.21–14.74), urinary tract infection during pregnancy (aOR = 4.20, 95% CI: 2.13–8.27), foul-smelling vaginal discharge (aOR = 3.22, 95% CI: 1.48–6.97), traditional herb use (aOR = 2.19, 95% CI: 1.16–4.13) and no formal education (aOR = 6.97, 95% CI: 1.50–32.46). Earlier gestational age, prolonged labour and primary education were also associated with culture positivity. Positive aerobic placental basal plate cultures were common in this population. These findings support improved recognition and management of maternal infection during antenatal and intrapartum care. However, culture positivity alone cannot distinguish invasive infection from colonization or contamination, and prospective studies are needed to clarify temporal and causal relationships.

## Introduction

Preterm birth, defined as delivery before 37 completed weeks of gestation, remains a major global public health challenge. An estimated 13.4 million babies were born preterm in 2020, with the highest burden occurring in low- and middle-income countries, particularly in sub-Saharan Africa [1]. Complications of prematurity are the leading cause of death among children under five years of age and are associated with long-term respiratory, neurological, visual, and developmental impairments among survivors [1–3].

Ascending bacterial infection of the uteroplacental tissues is a well-established pathway leading to spontaneous preterm labor. Microorganisms originating from the lower genital tract may invade the decidua, fetal membranes, placenta, and placental bed, triggering inflammatory responses characterized by cytokine release, prostaglandin production, cervical remodeling, and uterine contractions that culminate in preterm delivery [3,4]. Intrauterine bacterial infection has been implicated in up to 40% of spontaneous preterm births, particularly at earlier gestational ages [3]. The placental bed, which forms the maternal interface with the placenta, therefore represents an important site for investigating infection-related preterm labor.

The placenta ordinarily serves as a protective barrier against microbial invasion. However, bacterial, viral, and protozoal infections of placental tissues can disrupt placental function and contribute to spontaneous abortion, fetal growth restriction, stillbirth, congenital anomalies, preterm birth, neonatal morbidity, and neonatal mortality [5].

Previous studies have reported considerable variation in the prevalence of placental bacterial infection, ranging from approximately 20% to nearly 50% depending on the study population and diagnostic methods. A Kenyan study reported a prevalence of 47.4% among women with preterm births, while studies from other settings have documented prevalence estimates between 20% and 40% [6–8]. Maternal conditions such as prolonged rupture of membranes, urinary tract infection, HIV infection, and limited access to quality antenatal care have consistently been associated with an increased risk of ascending bacterial invasion and adverse pregnancy outcomes [9,10].

Uganda continues to face a substantial burden of preterm birth, with approximately 14% of babies born before 37 completed weeks of gestation. Prematurity remains one of the leading causes of neonatal illness and death, emphasizing the need for effective strategies to prevent and manage its underlying causes [1,11]. At Mubende Regional Referral Hospital, the number of preterm births increased from 1,119 in the 2022/2023 financial year to 1,417 in 2023/2024 (unpublished hospital records), yet little is known about the contribution of aerobic placental culture positivity to this increasing burden. Although previous studies have examined clinical chorioamnionitis and maternal risk factors for preterm birth, evidence on the prevalence of positive aerobic placental basal plate cultures and their associated factors remains limited in Uganda. This lack of evidence hampers efforts to identify women at increased risk, strengthen infection prevention during pregnancy, and develop targeted interventions to reduce infection-related preterm births. Therefore, this study aimed to estimate the prevalence of positive aerobic bacterial cultures from placental basal plate tissue and identify maternal factors associated with culture positivity among women with spontaneous preterm births at Mubende Regional Referral Hospital, Uganda.

## Materials and methods

### Ethics statement

Ethical approval for this study was obtained from the Kampala International University Research Ethics Committee under approval number KIU-2025–846. Administrative permission to conduct the research was granted by Mubende Regional Referral Hospital. Written informed consent was obtained from all participants after the study objectives, procedures, potential risks, and benefits had been clearly explained in either English or Luganda, depending on the participant’s preferred language. Participation was entirely voluntary, and only individuals aged 18 years or older who demonstrated the capacity to make informed decisions were enrolled. Each participant provided consent personally, and confidentiality was maintained through the use of unique study identification codes and secure storage of study records. The study was conducted in accordance with the ethical principles of the Declaration of Helsinki.

### Study design and setting

This hospital-based analytical cross-sectional study was conducted among women with preterm births at Mubende Regional Referral Hospital (MRRH), Uganda, from 15th May 2025–15th September 2025. The study sought to determine the prevalence of positive aerobic placental basal plate cultures and identify associated maternal, obstetric, and clinical factors. Mubende Regional Referral Hospital is a 400-bed government-owned facility located in the central business district of Mubende Town, approximately 150 km west of Mulago National Referral Hospital and 148 km east of Fort Portal Regional Referral Hospital. The hospital serves as the principal referral center for Mubende District and neighboring districts, including Kyankwanzi, Mityana, Kiboga, and Kassanda, providing comprehensive emergency obstetric and newborn care services to a predominantly rural population.

According to hospital records, 1,119 preterm births were recorded during the 2022/2023 financial year, increasing to 1,417 in 2023/2024. Between February and July 2023, approximately 300 women were managed for spontaneous preterm labor at the facility, underscoring the substantial burden of prematurity in the region.

### Study population

The study population comprised women with spontaneous preterm births (26^0/7–36^6/7 weeks of gestation) who delivered at Mubende Regional Referral Hospital during the study period. Eligible participants were women aged 18 years and above who provided written informed consent and had placental tissue available for specimen collection immediately after delivery. Women with medically indicated preterm deliveries due to major fetal congenital anomalies or conditions requiring immediate placental disposal were excluded. Participants with incomplete clinical records (including ANC card) or inadequate placental specimens for microbiological analysis were also excluded from the study.

### Sample size determination

Sample size was calculated separately for the study objectives. For objective one (prevalence of positive aerobic placental basal plate cultures), the Kish–Leslie formula for a single proportion was applied using a prevalence of 47.4% reported in a previous Kenyan study among women with preterm labor [7], yielding a minimum sample size of 383 participants. For objective two (associated factors), OpenEpi was used to estimate the required sample size for comparing two proportions, which produced a smaller sample than that obtained for objective one. Consequently, the larger sample size of 383 participants was adopted. All eligible women were enrolled consecutively during the study period, and the final sample comprised 383 participants.

### Sampling technique

Participants were enrolled consecutively from women with preterm births at Mubende Regional Referral Hospital until the required sample size of 383 was attained. Eligible women were those delivering between 26^0/7 and 36^6/7 weeks of gestation who provided written informed consent. Enrollment was conducted irrespective of ethnicity, religion, marital status, or other sociodemographic characteristics. This approach ensured that all eligible women presenting during the study period had an equal opportunity to participate.

### Data collection procedure

Eligible women were invited to participate after initial clinical assessment and stabilization. Whenever feasible, written informed consent was obtained before delivery. Women requiring urgent obstetric care were approached only after receiving the necessary treatment and becoming clinically stable enough to make an informed decision. The study objectives and procedures were explained in English or Luganda, according to participant preference. No study-specific specimen was collected before written informed consent had been obtained, and declining participation did not affect the care provided.

After enrolment, a structured interviewer-administered questionnaire was used to collect sociodemographic, obstetric, behavioral, and clinical information, including maternal age, educational level, HIV status, history of urinary tract infection, traditional herb use, and obstetric complications. Following delivery, placental tissue specimens were collected aseptically by trained clinicians and transported in sterile, appropriately labelled containers containing Stuart transport medium to the microbiology laboratory for bacterial culture and identification. Women who declined participation or from whom an adequate placental tissue specimen could not be obtained were excluded from the final analysis.

### Specimen collection and bacterial identification

Immediately after delivery, the placenta was placed with the maternal surface facing upward on a sterile receptacle. Using a new sterile scalpel and forceps for each participant, an approximately 1 × 1 cm full-thickness tissue specimen was obtained from the central maternal placental surface, away from the placental margins, fetal membranes and visibly contaminated areas. The specimen was immediately placed in a sterile, labelled container containing Stuart transport medium and transported to the microbiology laboratory within four hours of collection.

In the laboratory, specimens were processed using standard aerobic bacteriological culture methods. Bacterial isolates were identified using colony morphology, Gram-staining characteristics and conventional biochemical tests available in the study laboratory. Culture results were classified as positive when aerobic bacterial growth was detected and negative when no growth was detected. The distribution of recovered isolates was summarized descriptively using frequencies and percentages, with the total number of isolates as the denominator. The primary analyses focused on the prevalence of positive aerobic placental basal plate cultures and associated maternal factors.

### Study variables

The independent variables included sociodemographic, behavioral, obstetric, and clinical characteristics that could potentially influence the occurrence of aerobic placental culture positivity among women with spontaneous preterm labor. Sociodemographic variables included maternal age, educational level, and gravidity. Behavioral factors comprised alcohol consumption, smoking, recreational drug use, and the use of traditional herbs during pregnancy. Obstetric and clinical variables included gestational age at delivery, prolonged labor, duration of membrane rupture, history of preterm birth, urinary tract infection during pregnancy, intrauterine fetal death, antepartum hemorrhage, body mass index, HIV status, fever, foul-smelling vaginal discharge, and abdominal pain or uterine tenderness. Women with uncertain gestational age and no first-trimester ultrasound confirmation were excluded and were therefore not considered in the analysis.

The dependent variable was aerobic placental basal plate culture positivity. Participants whose placental basal plate specimens yielded aerobic bacterial growth were classified as culture positive, whereas those whose specimens yielded no bacterial growth were classified as culture negative.

### Data quality control

To ensure the reliability and credibility of findings on aerobic placental culture positivity and its associated factors, strict adherence to the inclusion and exclusion criteria was maintained throughout the study. The structured questionnaire was administered in English or the local language according to participant preference. Prior to the main study, the questionnaire was pre-tested among 10% of the target population, and necessary revisions were made to improve clarity, consistency, and completeness. The principal investigator trained the research assistants and supervised all study procedures to ensure standardized data collection and compliance with ethical requirements.

Placental bed tissue specimens were collected under strict aseptic conditions by trained healthcare professionals, labelled using unique participant identification codes, and transported in Stuart transport medium to the Mubende Regional Referral Hospital microbiology laboratory within four hours of collection. Standard operating procedures were followed during specimen handling, processing, and bacterial identification to minimize contamination and ensure consistency. Data collection forms were reviewed daily for completeness and accuracy, and all records were securely stored to maintain data integrity throughout the study.

### Data management and analysis

Data were coded and entered into Microsoft Excel before being exported to Stata version 17 for analysis. Participants’ sociodemographic, obstetric, behavioural and clinical characteristics were summarized using frequencies and percentages for categorical variables and means with standard deviations or medians with interquartile ranges for continuous variables, as appropriate.

The prevalence of positive aerobic placental basal plate cultures was calculated as the proportion of participants whose specimens yielded bacterial growth and reported with a 95% confidence interval. Associations between participant characteristics and culture positivity were initially examined using univariable logistic regression. Variables with p < 0.20 at univariable analysis and those considered biologically plausible were considered for inclusion in the multivariable logistic regression model. Adjusted odds ratios with corresponding 95% confidence intervals were reported, and p < 0.05 was considered statistically significant.

Multicollinearity in the final model was assessed using variance inflation factors (VIFs). Because fever, abdominal pain or uterine tenderness and foul-smelling vaginal discharge could represent overlapping manifestations of an infection-related clinical syndrome, their pairwise co-occurrence was examined using 2 × 2 cross-tabulations and quantified using phi coefficients. The mean VIF for the final model was 1.51, while the individual VIFs were 1.06 for fever, 1.10 for abdominal pain or uterine tenderness and 1.06 for foul-smelling vaginal discharge. A composite indicator was generated to identify culture-negative women who had at least one of the following clinical findings: fever, abdominal pain or uterine tenderness, or foul-smelling vaginal discharge. The number of findings present in each culture-negative woman was also summarized to account for overlap.

Biologically plausible interactions between HIV status and history of urinary tract infection and between membrane-rupture duration and prolonged labour were assessed by adding product terms separately to the fully adjusted model while retaining the corresponding main effects and all other covariates. Because membrane-rupture duration had three categories, its interaction with prolonged labour was evaluated using a joint two-degree-of-freedom Wald test. Interaction terms with p < 0.05 were considered statistically significant.

The multivariable analysis was exploratory, and no formal adjustment for multiple testing was applied. Consequently, the reported p-values are nominal and should be interpreted alongside the adjusted effect estimates and their 95% confidence intervals rather than as definitive confirmatory evidence.

## Results

### Characteristics of the study participants

A total of 412 women with spontaneous preterm births were screened for eligibility during the study period. Of these, 21 did not meet the inclusion criteria, while 8 declined participation. Consequently, 383 women provided written informed consent and were included in the final analysis (Fig 1).

**Fig 1:**
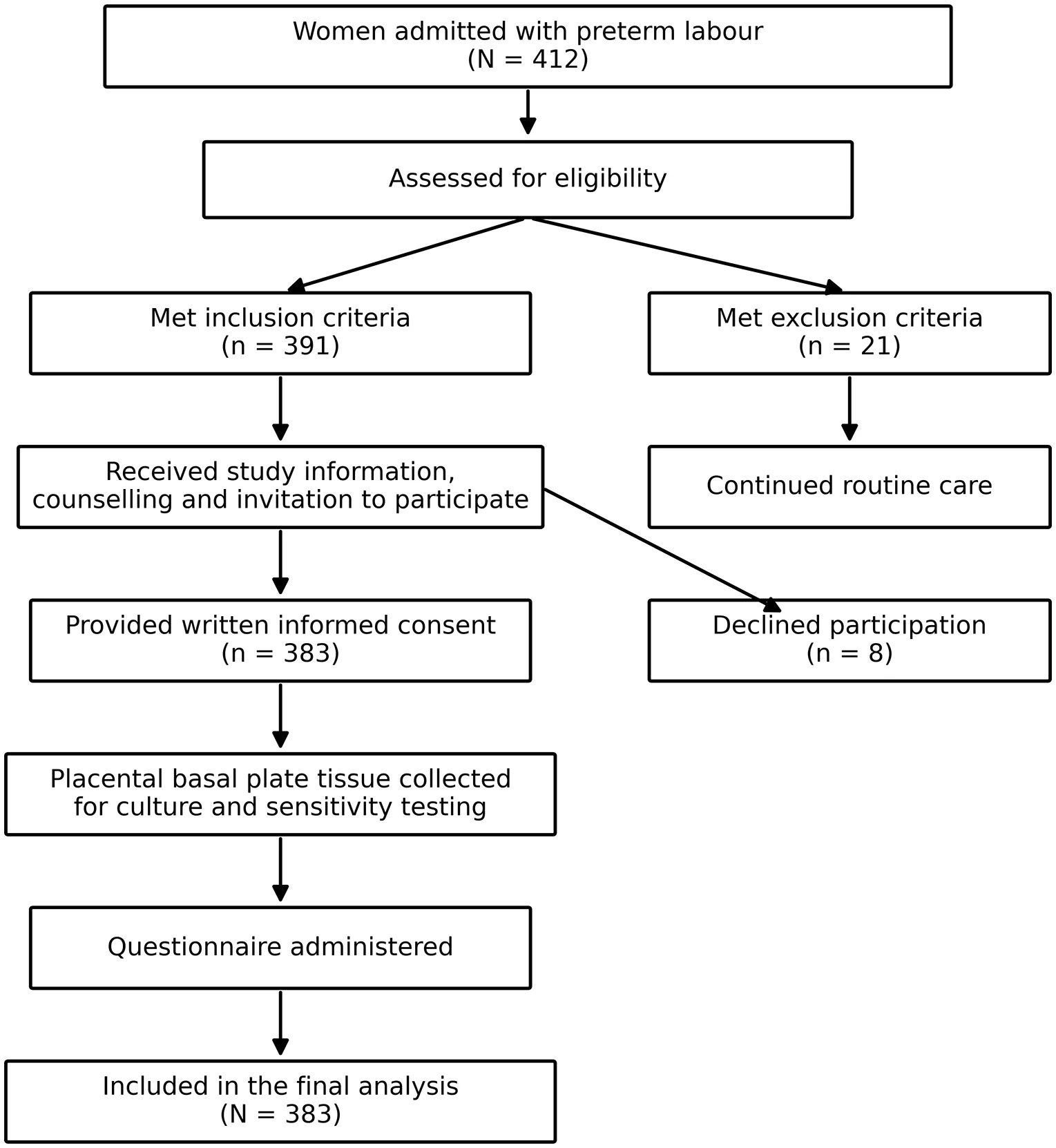
Participant flow diagram. Flow of women assessed for eligibility, excluded, invited to participate, enrolled and included in the final analysis.

More than half of the participants were aged 20–29 years (54.1%, *n* = 207), and 52.2% (*n* = 200) had attained primary-level education. Nearly half of the women (44.9%, *n* = 172) had experienced two to three pregnancies, while 54.3% (*n* = 208) delivered between 33 and 36 weeks of gestation.

Most participants did not experience prolonged labor (80.4%, *n* = 308), and 69.2% (*n* = 265) delivered within 12 hours of membrane rupture. A history of preterm birth was reported by 20.1% (*n* = 77), whereas 25.1% (*n* = 96) had experienced a urinary tract infection during the current pregnancy. The majority of women had a normal body mass index (49.9%, *n* = 191), and 5.0% (*n* = 19) were living with HIV. Regarding behavioral and clinical characteristics, alcohol consumption was reported by 15.7% (*n* = 60), smoking by 3.1% (*n* = 12), and recreational drug use by 2.1% (*n* = 8). Traditional herbs had been used during pregnancy by 28.7% (*n* = 110). Fever, foul-smelling vaginal discharge, and abdominal pain or uterine tenderness were reported by 18.5% (*n* = 71), 17.0% (*n* = 65), and 23.5% (*n* = 90) of participants, respectively. Detailed participant characteristics are presented in Table 1.

**Table 1:** Sociodemographic, Obstetric, and Clinical Characteristics of Study Participants (N = 383).

Characteristic	Frequency (n)	Percentage (%)
**Age (yrs)**		
<20	61	15.9
20-29	207	54.1
30-39	96	25.1
≥40	19	5.0
**Education Level**		
None	40	10.4
Primary	200	52.2
Secondary	114	29.8
Tertiary	29	7.6
**Gravidity**		
1	96	25.1
2-3	172	44.9
≥4	115	30.0
**Gestational Age**		
<28 weeks	40	10.4
28weeks 0 day-32 weeks 6days	135	35.2
33weeks 0 day-36 weeks 6days	208	54.3
**Prolonged Labor (>12 hrs)**		
No	308	80.4
Yes	75	19.6
**Membrane Rupture to Delivery**		
<12 hours	265	69.2
12-24 hours	78	20.4
>24 hours	40	10.4
**History of Preterm Birth**		
No	306	79.9
Yes	77	20.1
**History of UTI in Pregnancy**		
No	287	74.9
Yes	96	25.1
**History of IUFD**		
No	348	90.9
Yes	35	9.1
**History of APH**		
No	328	85.6
Yes	55	14.4
**BMI Category**		
Underweight	31	8.1
Normal	191	49.9
Overweight	115	30.0
Obese	46	12.0
**HIV Status**		
Negative	364	95.0
Positive	19	5.0
**Smoking**		
No	371	96.9
Yes	12	3.1
**Alcohol Use**		
No	323	84.3
Yes	60	15.7
**Recreational Drug Use**		
No	375	97.9
Yes	8	2.1
**Use of Traditional Herbs**		
No	273	71.3
Yes	110	28.7
**Fever**		
No	312	81.5
Yes	71	18.5
**Foul-smelling vaginal discharge**		
No	318	83.0
Yes	65	17.0
**Abdominal Pain/Tenderness**		
No	293	76.5
Yes	90	23.5

yrs, years; IUFD, intrauterine fetal death; BMI, body mass index.

APH ante-partum hemorrhage, UTI urinary tract infection.

### Prevalence of positive aerobic placental basal plate cultures

Among the 383 women included in the study, 159 had positive aerobic placental basal plate cultures, corresponding to an overall prevalence of 41.5% (95% CI: 36.5%–46.6%). The distribution of aerobic placental culture results is illustrated in Fig 2.

**Fig 2:**
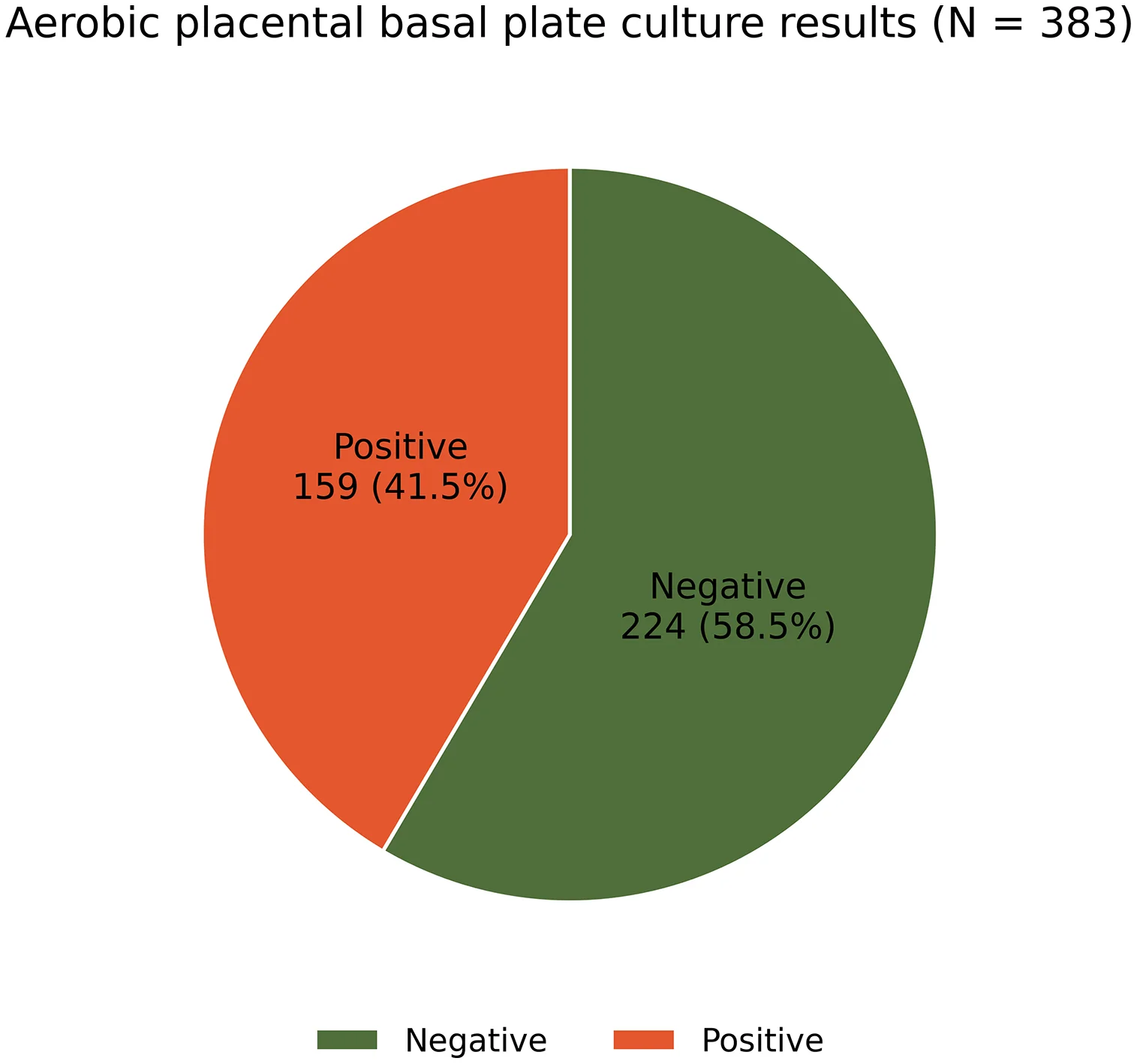
Aerobic placental basal plate culture results. Distribution of positive and negative aerobic bacterial culture results among 383 women with spontaneous preterm births.

Clinical findings potentially suggestive of maternal infection were also present among some women with negative aerobic cultures. Of the 224 culture-negative women, 16 (7.1%) had fever, 20 (8.9%) had abdominal pain or uterine tenderness and 20 (8.9%) had foul-smelling vaginal discharge. After accounting for overlap, 54 culture-negative women (24.1%) had at least one of these findings. Fifty-two had exactly one finding, while two had both abdominal pain or uterine tenderness and foul-smelling vaginal discharge. None of the culture-negative women had all three findings.

A total of 184 aerobic bacterial isolates were recovered from the 159 culture-positive placental basal plate specimens. Gram-negative organisms accounted for 141 isolates (76.6%), while gram-positive organisms accounted for 43 (23.4%). The most frequently recovered organisms were Escherichia coli (94/184, 51.1%), Klebsiella pneumoniae (45/184, 24.5%) and Staphylococcus aureus (40/184, 21.7%). The remaining organisms are presented in Table 2.

**Table 2:** Distribution of aerobic bacterial isolates recovered from culture-positive placental basal plate specimens (N = 184 isolates).

Bacterial isolate	Frequency, n	Percentage of isolates, %
**Gram-negative bacteria**	**141**	**76.6**
*Escherichia coli*	94	51.1
*Klebsiella pneumoniae*	45	24.5
*Aeromonas hydrophila*	1	0.5
*Pseudomonas putida*	1	0.5
**Gram-positive bacteria**	**43**	**23.4**
*Staphylococcus aureus*	40	21.7
*Enterococcus faecalis*	2	1.1
Group B *Streptococcus*	1	0.5
**Total isolates**	**184**	**100.0**

*Percentages were calculated using the 184 recovered isolates as the denominator. These isolates were recovered from 159 culture-positive specimens, indicating that some specimens yielded more than one organism*.

### Factors associated with aerobic placental culture positivity

In the bivariable analysis, several variables met the pre-specified threshold of *p* < 0.20 and were considered for inclusion in the multivariable model. These included educational level, gestational age at delivery, prolonged labor, duration from membrane rupture to delivery, HIV status, history of urinary tract infection (UTI) during pregnancy, use of traditional herbs, fever, foul-smelling vaginal discharge, and abdominal pain or uterine tenderness (Table 3).

**Table 3:** Bivariable Analysis of Factors Associated with Aerobic Placental Culture Positivity (N = 383).

Factor	Culture negative (n = 224)	Culture positive (n = 159)	Crude OR (95% CI)	p-value
	n (%)	n (%)		
**Age**				
≥40	10 (4.5)	9 (5.7)	1.00	
<20	32 (14.3)	29 (18.2)	1.01 (0.36 - 2.82)	0.990
20-29	123 (54.9)	84 (52.8)	0.76 (0.30 - 1.95)	0.566
30-39	59 (26.3)	37 (23.3)	0.70 (0.26 - 1.88)	0.474
**Education Level**				
Tertiary	23 (10.3)	6 (3.8)	1.00	
Secondary	90 (40.2)	24 (15.1)	1.02 (0.37 - 2.79)	0.966
Primary	96 (42.9)	104 (65.4)	4.15 (1.62 - 10.63)	**0.003**
None	15 (6.7)	25 (15.7)	6.39 (2.12 - 19.25)	**0.001**
**Gravidity**				
1	56 (25.0)	40 (25.2)	1.00	
2-3	101 (45.1)	71 (44.7)	0.98 (0.59 - 1.63)	0.951
≥4	67 (29.9)	48 (30.2)	1.00 (0.58 - 1.74)	0.992
**Gestational Age**				
33-36 wks	136 (60.7)	72 (45.3)	1.00	
<28 wks	18 (8.0)	22 (13.8)	2.31 (1.16 - 4.58)	**0.017**
28-32 wks	70 (31.3)	65 (40.9)	1.75 (1.13 - 2.73)	**0.013**
**Prolonged Labor**				
No	198 (88.4)	110 (69.2)	1.00	
Yes	26 (11.6)	49 (30.8)	3.39 (2.00 - 5.76)	**<0.001**
**Membrane Rupture**				
<12 hrs	166 (74.1)	99 (62.3)	1.00	
12-24 hrs	50 (22.3)	28 (17.6)	0.94 (0.56 - 1.59)	0.814
>24 hrs	8 (3.6)	32 (20.1)	6.71 (2.97 - 15.13)	**<0.001**
**BMI**				
Normal	112 (50.0)	79 (49.7)	1.00	
Underweight	18 (8.0)	13 (8.2)	1.02 (0.47 - 2.21)	0.952
Overweight	68 (30.4)	47 (29.6)	0.98 (0.61 - 1.57)	0.933
Obese	26 (11.6)	20 (12.6)	1.09 (0.57 - 2.09)	0.794
**HIV Status**				
Negative	216 (96.4)	148 (93.1)	1.00	
Positive	8 (3.6)	11 (6.9)	2.01 (0.79 - 5.11)	0.144
**History of Preterm Birth**				
No	193 (86.2)	113 (71.1)	1.00	
Yes	31 (13.8)	46 (28.9)	2.53 (1.52 - 4.23)	**<0.001**
**History of UTI**				
No	190 (84.8)	97 (61.0)	1.00	
Yes	34 (15.2)	62 (39.0)	3.57 (2.20 - 5.80)	**<0.001**
**History of IUFD**				
No	205 (91.5)	143 (89.9)	1.00	
Yes	19 (8.5)	16 (10.1)	1.21 (0.60 - 2.43)	0.597
**History of APH**				
No	194 (86.6)	134 (84.3)	1.00	
Yes	30 (13.4)	25 (15.7)	1.21 (0.68 - 2.14)	0.522
**Smoking**				
No	217 (96.9)	154 (96.9)	1.00	
Yes	7 (3.1)	5 (3.1)	1.01 (0.31 - 3.23)	0.991
**Alcohol Use**				
No	189 (84.4)	134 (84.3)	1.00	
Yes	35 (15.6)	25 (15.7)	1.01 (0.58 - 1.76)	0.979
**Recreational Drug Use**				
No	219 (97.8)	156 (98.1)	1.00	
Yes	5 (2.2)	3 (1.9)	0.84 (0.20 - 3.58)	0.816
**Use of Herbs**				
No	175 (78.1)	98 (61.6)	1.00	
Yes	49 (21.9)	61 (38.4)	2.22 (1.42 - 3.49)	**<0.001**
**Fever**				
No	208 (92.9)	104 (65.4)	1.00	
Yes	16 (7.1)	55 (34.6)	6.88 (3.76 - 12.58)	**<0.001**
**Foul-smelling Discharge**				
No	204 (91.1)	114 (71.7)	1.00	
Yes	20 (8.9)	45 (28.3)	4.03 (2.27 - 7.15)	**<0.001**
**Abdominal Pain**				
No	204 (91.1)	89 (56.0)	1.00	
Yes	20 (8.9)	70 (44.0)	8.02 (4.60 - 13.99)	**<0.001**

Note: Variables with p < 0.20 were considered for multivariable analysis. wks, weeks; CI, confidence interval; cOR, crude odds ratio; yrs, years; IUFD, intrauterine fetal death; BMI, body mass index; APH, antepartum hemorrhage; UTI, urinary tract infection.

Pairwise analysis demonstrated limited overlap among the three clinical signs. Fever and abdominal pain or uterine tenderness co-occurred in 20 women (5.2% of the total sample; φ = 0.053), fever and foul-smelling vaginal discharge in 17 women (4.4%; φ = 0.089), and abdominal pain or uterine tenderness and foul-smelling vaginal discharge in 24 women (6.3%; φ = 0.143). The individual VIFs were 1.06 for fever, 1.10 for abdominal pain or uterine tenderness and 1.06 for foul-smelling vaginal discharge. These findings indicated limited pairwise association and no problematic multicollinearity among the three predictors.

There was no statistical evidence of interaction between HIV status and history of urinary tract infection (interaction coefficient = −1.57; ratio of odds ratios = 0.21, 95% CI: 0.01–3.07; p = 0.253). Similarly, the interaction between membrane-rupture duration and prolonged labour was not statistically significant. The interaction estimates were 2.64 (95% CI: 0.24–28.94; p = 0.427) for rupture lasting 12–24 hours and 1.07 (95% CI: 0.08–14.59; p = 0.961) for rupture lasting more than 24 hours, relative to less than 12 hours. The joint Wald test was not significant (χ^2^ [2] = 0.63; p = 0.728). The interaction terms were therefore not retained in the final main-effects model.

The multivariable logistic regression model demonstrated good fit (Hosmer–Lemeshow test, *p* = 0.208) and showed no evidence of multicollinearity (mean variance inflation factor = 1.51). After adjustment for potential confounders, several factors remained independently associated with culture positivity (Table 4). Women with no formal education had nearly sevenfold higher odds of culture positivity compared with those who had attained tertiary education (aOR = 6.97, 95% CI: 1.50–32.46), while primary education was associated with more than threefold increased odds (aOR = 3.57, 95% CI: 1.05–12.11).

**Table 4:** Multivariable Logistic Regression of Factors Associated with Aerobic Placental Culture Positivity (N = 383).

Factor	cOR (95% CI)	p-value	aOR	95% CI	p-value
**Education Level**					
Tertiary	1.00		1.00		
None	6.39 (2.12 - 19.25)	0.001	6.97	1.50 - 32.46	**0.013**
Primary	4.15 (1.62 - 10.63)	0.003	3.57	1.05 - 12.11	**0.041**
Secondary	1.02 (0.37 - 2.79)	0.966	0.73	0.20 - 2.72	**0**.641
**Gestational Age**					
33-36 wks	1.00		1.00		
<28 weeks	2.31 (1.16 - 4.58)	0.017	2.88	1.08 - 7.70	**0.035**
28-32 weeks	1.75 (1.13 - 2.73)	0.013	2.11	1.10 - 4.04	**0.024**
**Prolonged Labor**					
No	1.00		1.00		
Yes	3.39 (2.00 - 5.76)	<0.001	2.68	1.25 - 5.75	**0.011**
**Membrane Rupture**					
<12 hrs	1.00		1.00		
12-24 hrs	0.94 (0.56 - 1.59)	0.814	0.80	0.37 - 1.72	0.569
>24 hrs	6.71 (2.97 - 15.13)	<0.001	5.93	2.08 - 16.89	**0.001**
**HIV Positive Status**					
Negative	1.00		1.00		
Positive	2.01 (0.79 - 5.11)	0.144	4.23	1.21 - 14.74	**0.024**
**History of UTI in Pregnancy**					
No	1.00		1.00		
Yes	3.57 (2.20 - 5.80)	<0.001	4.20	2.13 - 8.27	**<0.001**
**History of Preterm Birth**					
No	1.00		1.00		
Yes	2.53(1.52-4.23)	<0.001	1.56	0.74-3.15	0.243
**Use of Traditional Herbs**					
No	1.00		1.00		
Yes	2.22 (1.42 - 3.49)	<0.001	2.19	1.16 - 4.13	**0.016**
**Fever**					
No	1.00		1.00		
Yes	6.88 (3.76 - 12.58)	<0.001	11.32	4.89 - 26.19	**<0.001**
**Foul-smelling vaginal discharge**					
No	1.00		1.00		
Yes	4.03 (2.27 - 7.15)	<0.001	3.22	1.48 - 6.97	**0.003**
**Abdominal Pain/Tenderness**					
No	1.00		1.00		
Yes	8.02 (4.60 - 13.99)	<0.001	9.13	4.37 - 19.08	**<0.001**

CI confidence Interval, cOR crude odds ratio, aOR adjusted odds ratio

Clinical factors showed the strongest associations. Fever increased the odds of culture positivity more than elevenfold (aOR = 11.32, 95% CI: 4.89–26.19), and abdominal pain or uterine tenderness was associated with a ninefold increase in odds (aOR = 9.13, 95% CI: 4.37–19.08). Women with membrane rupture lasting more than 24 hours were almost six times more likely to have a positive aerobic placental basal plate culture (aOR = 5.93, 95% CI: 2.08–16.89), while a history of UTI during pregnancy (aOR = 4.20, 95% CI: 2.13–8.27) and HIV-positive status (aOR = 4.23, 95% CI: 1.21–14.74) were also significantly associated factors.

Deliveries occurring before 28 weeks (aOR = 2.88, 95% CI: 1.08–7.70) and between 28 and 32 weeks of gestation (aOR = 2.11, 95% CI: 1.10–4.04) were associated with increased odds of culture positivity compared with deliveries at 33–36 weeks. In addition, prolonged labor (aOR = 2.68, 95% CI: 1.25–5.75), the use of traditional herbs during pregnancy (aOR = 2.19, 95% CI: 1.16–4.13), and foul-smelling vaginal discharge (aOR = 3.22, 95% CI: 1.48–6.97) remained independently associated with culture positivity.

## Discussion

In this study, 41.5% of placental basal plate tissue specimens from women with spontaneous preterm births yielded positive aerobic bacterial cultures. Culture positivity was associated with several maternal clinical, obstetric and socioeconomic characteristics. These findings should be interpreted cautiously: the analyses were exploratory, and without adjustment for multiple testing, some associations may have occurred by chance. Because specimens were collected after delivery within a cross-sectional study, we could not determine whether bacterial presence preceded labour or contributed to preterm birth. Positive cultures alone also cannot distinguish invasive infection from colonization or contamination. Further studies are needed to confirm these associations and clarify their clinical significance.

The prevalence of positive placental tissue bacterial cultures in this study was 41.5%, indicating that bacterial colonization or culture positivity of placental tissue was common among women with preterm births. This estimate is numerically similar to the 47.4% reported by Kibunja in Kenya, although differences in study populations, specimen collection and diagnostic methods limit direct comparison [7]. Similar observations have been documented in studies linking microbial invasion of placental tissues with spontaneous preterm labor and adverse neonatal outcomes [3,12]. In contrast, lower prevalences have been reported in some high-income settings, including 27% by Stout et al and approximately 20% in other hospital-based studies, likely reflecting differences in antenatal care utilization, early infection screening, and broader socioeconomic determinants of maternal health [8,13]

Variations in reported prevalence should be interpreted within the context of the diagnostic approaches employed. The present study used aerobic bacterial culture, which detects viable aerobic bacterial growth but cannot by itself distinguish infection from colonization or contamination. Histopathological studies, by comparison, detect inflammatory changes such as chorioamnionitis that may arise from bacterial, viral, anaerobic, or even sterile inflammatory processes This supports the widely accepted concept [14,15]. Molecular techniques, including bacterial PCR and microbiome sequencing, frequently identify bacterial DNA irrespective of microbial viability and often report substantially higher rates of placental colonization [16–18]. Consequently, the prevalence observed in this study represents the prevalence of aerobic culture positivity; however, culture findings alone cannot establish invasive placental infection or causation in preterm birth [3,19]. Although several aerobic bacterial species were recovered, organism-specific interpretation and antimicrobial susceptibility patterns were outside the scope of the present analysis, which focused on the prevalence of culture positivity and associated maternal factors.

Several clinical manifestations remained independently associated with culture positivity after adjustment for potential confounders. Maternal fever was associated with more than elevenfold higher odds of culture positivity, while abdominal pain or uterine tenderness increased the likelihood nearly ninefold. These findings are consistent with the classical clinical presentation of intra-amniotic infection and chorioamnionitis [20,21]. Fever and uterine tenderness were independently associated with culture positivity in this study. Fever reflects systemic activation of the host inflammatory response, whereas uterine tenderness results from localized intrauterine inflammation characterized by cytokine production and increased prostaglandin synthesis within the decidua, fetal membranes, and myometrium, ultimately promoting uterine contractility and preterm labor [22,23]. Likewise, foul-smelling vaginal discharge remained a significant predictor, supporting previous evidence that abnormal genital tract colonization frequently precedes ascending intrauterine infection and subsequent placental involvement [24,25]

Among obstetric factors, prolonged rupture of membranes exceeding 24 hours was one of the strongest factors associated with culture positivity. This observation aligns with the established understanding that intact fetal membranes constitute a critical barrier against ascending microorganisms from the lower genital tract [10,26]. Once membrane integrity is compromised, pathogens gain direct access to the uterine environment, substantially increasing the risk of microbial invasion and inflammation. Similarly, prolonged labor independently increased the likelihood of culture positivity, likely reflecting sustained exposure to ascending bacteria and repeated obstetric manipulations during labor [27,28]. These findings underscore the importance of timely obstetric interventions and strict adherence to infection-prevention practices during labor management.

Earlier gestational age at delivery was also associated with significantly higher odds of culture positivity. Women delivering before 28 weeks and those delivering between 28 and 32 weeks were more likely to have culture positivity than those delivering between 33 and 36 weeks. This association is compatible with previous research linking intrauterine infection to earlier spontaneous preterm birth, but the timing and causal direction of the association cannot be determined from the present study [3,29]. Microbial invasion stimulates inflammatory cascades involving interleukins, tumor necrosis factor, and prostaglandins, ultimately triggering cervical ripening, membrane weakening, and uterine contractions that culminate in premature delivery [9,30]. Maternal health conditions also contributed substantially to the likelihood of culture positivity. Women with a history of urinary tract infection during pregnancy had more than fourfold increased odds of culture positivity. This finding is biologically plausible because uropathogenic organisms frequently colonize both the urinary and genital tracts, facilitating ascending infection into the uterine cavity [3,31]. Previous systematic reviews have consistently demonstrated that untreated bacteriuria and urinary tract infections increase the risk of preterm birth and adverse perinatal outcomes [32]. Routine antenatal screening and prompt treatment of urinary tract infections therefore remain essential preventive strategies.

HIV-positive women were also significantly more likely to have culture positivity. Similar findings have been reported among HIV-infected pregnant women, where persistent immune dysregulation and impaired mucosal defenses predispose to ascending bacterial infections and chorioamnionitis [33,34]. Emerging evidence further suggests that alterations in placental immune function among women living with HIV may influence susceptibility to microbial invasion and inflammatory injury [35]. Strengthening integrated antenatal and HIV services may therefore contribute to reducing infection-related obstetric complications in high-burden settings.

Beyond biological and clinical determinants, this study highlights the influence of social factors on the likelihood of culture positivity. Women with no formal education had nearly sevenfold higher odds of culture positivity, while those with only primary education remained significantly more vulnerable than women with tertiary education. Educational attainment likely serves as a proxy for broader social determinants, including health literacy, nutritional status, economic security, and access to quality antenatal care [36]. Lower educational levels may limit awareness of early symptoms of infection and reduce opportunities for timely healthcare utilization, thereby increasing vulnerability to adverse pregnancy outcomes.

The independent association between traditional herb use during pregnancy and culture positivity is particularly noteworthy. Herbal medicine remains deeply embedded within maternal healthcare practices across sub-Saharan Africa, with utilization rates ranging from 20% to more than 70% in some communities [36,37]. Non-sterile preparations or vaginal application of herbal remedies could potentially introduce microorganisms or alter the vaginal microbiota. However, the present study did not assess specific preparations, routes of administration or microbial contamination and therefore cannot establish these mechanisms. The association between traditional herb use and culture positivity remains exploratory and may reflect residual confounding or chance. Further research with detailed exposure assessment is needed before drawing conclusions about the safety of particular remedies or recommending changes to traditional practices.

Overall, the observed associations provide hypotheses for further investigation rather than establish targets for preventing preterm birth. Proposed biological and social explanations remain tentative because the relevant mechanisms were not directly evaluated. The study did not assess whether interventions addressing the associated characteristics would reduce culture positivity or improve pregnancy outcomes. Prospective studies incorporating contamination controls, placental histopathology and broader microbiological testing are needed to clarify the clinical significance of culture positivity and determine whether these associations are reproducible.

### Strengths and limitations

This study provides one of the first microbiologically confirmed assessments of positive placental tissue bacterial cultures and their associated factors among women with spontaneous preterm births in Uganda. The relatively large sample size, standardized data collection, and rigorous microbiological procedures enhanced the reliability of the findings. The inclusion of clinical, obstetric, and socioeconomic variables enabled a comprehensive assessment of factors associated with culture positivity.

Several limitations should be considered. The single-centre cross-sectional design limits generalizability and precludes causal inference. Conventional aerobic culture methods may have underestimated bacterial presence by failing to detect anaerobic, fastidious or non-cultivable organisms. Some exposures were self-reported and may be subject to recall bias, while residual confounding from unmeasured factors cannot be excluded. In addition, the mode of delivery was not recorded and could not be evaluated as a potential source of bacterial contamination or confounding. Finally, the absence of molecular diagnostics and placental histopathology limited the ability to distinguish true infection from bacterial colonization or contamination. Because several associations were examined without adjustment for multiple testing, the possibility of false-positive findings cannot be excluded. The reported significance levels are nominal, and the associations should therefore be interpreted as exploratory pending confirmation in independent studies. Although the prespecified interactions were not statistically significant, the study had limited power to detect effect modification within small joint-exposure subgroups. In particular, only five HIV-positive women reported a history of urinary tract infection, resulting in an imprecise HIV × UTI interaction estimate. Approximately one-quarter of women with negative aerobic cultures had at least one clinical finding potentially suggestive of infection. This may partly reflect the inability of aerobic culture to detect anaerobic, fastidious, low-density or non-cultivable organisms. However, fever, abdominal pain or uterine tenderness and foul-smelling vaginal discharge are not specific for intra-amniotic infection and may have noninfectious or extrauterine explanations. Therefore, these culture-negative women could not be classified as having confirmed placental infection on the basis of clinical findings alone.

## Conclusions and recommendations

Positive aerobic bacterial cultures were found in 41.5% of placental basal plate specimens from women with spontaneous preterm births at Mubende Regional Referral Hospital. Several maternal characteristics were associated with culture positivity after multivariable adjustment. However, these findings are exploratory, and some associations may reflect chance because no adjustment for multiple testing was applied. The cross-sectional design and sampling after delivery limit conclusions about whether bacterial presence contributed to preterm birth, while the diagnostic methods used cannot distinguish invasive infection from colonization or contamination. Prospective studies across multiple centers, using contamination controls and complementary diagnostic methods, are needed to confirm these findings and clarify their clinical significance.

## Supporting information

S1 Data De-identified participant-level minimal dataset. Dataset containing the participant-level values used to generate the descriptive statistics, figures and regression analyses reported in the manuscript.
(XLS)

## Abbreviations

AMR: antimicrobial resistance
APH: antepartum hemorrhage
aOR: adjusted odds ratio
BMI: body mass index
CI: confidence interval
CLSI: clinical and laboratory standards institute
cOR: crude odds ratio
HIV: human immunodeficiency virus
IUFD: intrauterine fetal death
KIU: Kampala international university
MRRH: Mubende regional referral hospital
UTI: urinary tract infection
WHO: World Health Organization
